# Cellulose-fueled extracellular electron transfer in termite gut microbes

**DOI:** 10.1128/aem.01272-26

**Published:** 2026-08-27

**Authors:** Xiao Deng, Akihiro Okamoto

**Affiliations:** 1 Research Center for Macromolecules and Biomaterials, National Institute for Materials Science 52747, Tsukuba, Ibaraki, Japan; 2 Graduate School of Chemical Sciences and Engineering, Hokkaido University 592380, Sapporo, Japan; 3 Graduate School of Science and Technology, University of Tsukuba 624301, Tsukuba, Ibaraki, Japan; 4 Research Center for Autonomous Systems Materialogy, Institute of Science Tokyo 163706, Yokohama, Kanagawa, Japan; Norwegian University of Life Sciences, Ås, Norway

**Keywords:** redox mediators, microbiome, gram-positive bacteria, cell wall permeability, transmission electron microscopy, bioelectrochemistry

## Abstract

Termites decompose billions of tons of lignocellulosic material annually through the cellulolytic metabolism of their gut microbiota. However, the mechanisms by which cellulose degradation drives anaerobic metabolism of the gut microbiome remain poorly understood. Here, we demonstrate cellulose-driven extracellular electron transfer (EET) in both a termite gut-derived gram-positive cellulolytic bacterium, *Ruminiclostridium cellobioparum* subsp. *termitidis* CT1112, and the termite gut microbiome. CT1112 cells produced current in the presence of cellulose or cellobiose as the electron donor under potentiostatic conditions. This activity was strongly dependent on self-secreted redox mediators. EET was markedly impaired when stationary-phase CT1112 developed a thickened, multilayered cell wall, reducing accessibility to exogenously added redox mediators, such as riboflavin, and small redox-active molecules, such as diaminobenzidine. As a proof of concept, current production from cellulose was also observed in the termite gut microbiome, together with increases in the relative abundances of Bacillota, Bacteroidota, and Pseudomonadota. These results support the presence of EET-capable bacteria in the termite gut and suggest that EET contributes to redox balance within the steep redox gradients of the gut. Our study expands the environmental relevance of EET in carbon cycling and highlights termite-derived microbiomes as promising platforms for bioelectrochemical applications, including cellulolytic microbial fuel cells.

**IMPORTANCE:**

Termites play major roles in carbon cycling in tropical ecosystems by decomposing lignocellulosic biomass with the help of their gut microbiota. However, the microbial physiology underlying this highly efficient cellulose conversion remains poorly understood. Here, we show that both a cellulolytic termite gut isolate and the termite gut microbiome are capable of extracellular electron transfer (EET) using cellulose and its derivatives as electron donors. These findings suggest that cellulose degradation in the termite gut is coupled to extracellular redox processes, expanding our understanding of how this globally important process proceeds in anaerobic environments. They also raise the possibility that EET contributes to redox homeostasis within the gut microenvironment and open new avenues for investigating the electrochemical physiology of termite gut microbes, including their ability to reduce dietary iron(III) minerals and their potential to participate in syntrophic interspecies electron transfer. Moreover, this work highlights termite-derived microbes as promising platforms for developing efficient cellulose-to-electricity conversion technologies.

## INTRODUCTION

Termites are among the most efficient cellulose degraders in nature, decomposing 3–7 billion tons of lignocellulosic materials annually, and are thus major drivers of global carbon cycling (1). Their superior cellulose-degrading capability is largely owing to their highly specialized gut microbiomes, which produce diverse cellulolytic enzymes (2). Cellulose is generally considered to be hydrolyzed into soluble oligosaccharides and sugars, including cellodextrins, cellobiose, and glucose, which are then further metabolized through glycolytic and fermentative pathways (3, 4). During these processes, excess reducing equivalents are generated and must be balanced for regeneration of oxidized nicotinamide adenine dinucleotide (NAD^+^) and to sustain microbial metabolism. Under anaerobic conditions, many environmental microbes have been shown to transfer excess electrons generated during metabolism to extracellular electron acceptors through a process known as extracellular electron transfer (EET) (5). However, whether and how EET proceeds during anaerobic cellulose degradation remain poorly understood (6, 7).

**Fig 1:**
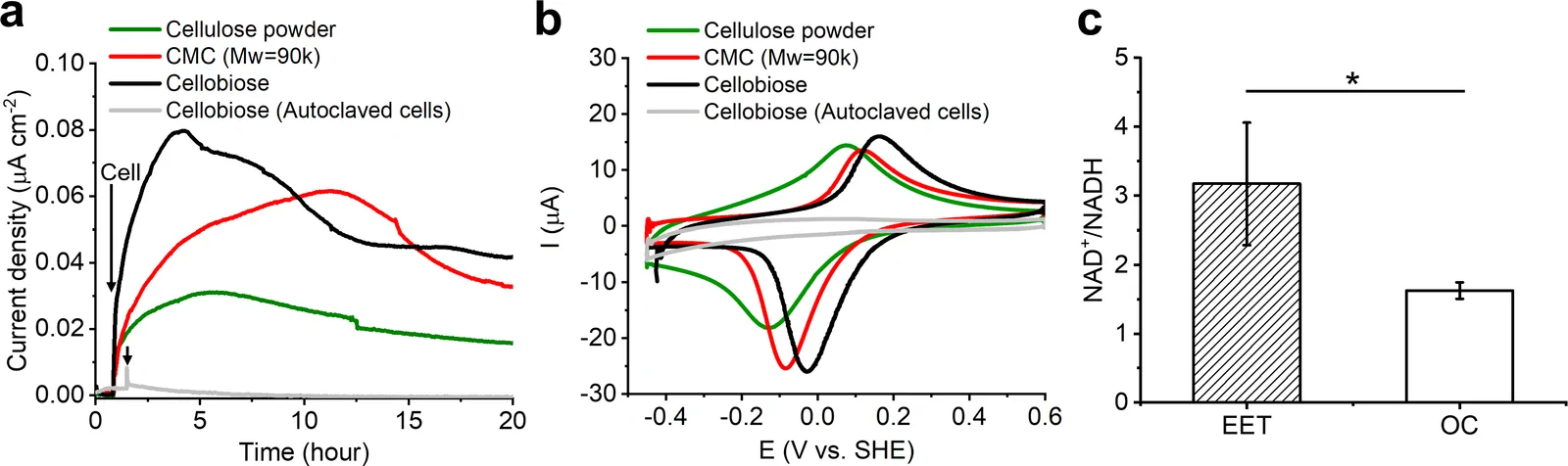
Extracellular electron transfer (EET) capability of the termite gut-derived cellulolytic gram-positive strain CT1112. (**a**) Representative anodic current curves produced by exponential-phase CT1112 cells supplied with different electron donors. CMC, carboxymethyl cellulose. Arrows indicate the time of cell inoculation into the reactors. (**b**) Cyclic voltammograms recorded following the current measurements, with anodic (oxidation) currents plotted as positive values and cathodic (reduction) currents plotted as negative values. (**c**) NAD^+^/NADH ratios of CT1112 cells after incubation in electrochemical reactors with cellobiose as the electron donor in the presence (EET) or absence (open circuit, OC) of a + 0.4 V-poised electrode. Data are shown as mean ± SD from three independent reactors (*n* = 3). *, *P* = 0.0459 by one-tailed Student’s *t*-test.

The termite gut is characterized by steep spatial redox gradients arising directly from intense microbial metabolism of cellulose hydrolysis products (Fig. 1a). Fermentation products such as acetate and hydrogen accumulate in the gut lumen during cellulose degradation but decline sharply toward the gut periphery due to host absorption and microbial consumption at the epithelium (8–10). Although oxygen diffuses inward from the gut wall, it is rapidly scavenged by epithelial-associated microbes, resulting in strictly anoxic conditions within 200–300 µm of the wall (11, 12). Consequently, redox potential within a single proctodeal segment can vary from approximately −200 mV to +200 mV (13, 14), reflecting pronounced electrochemical heterogeneity over submillimeter scales. This spatial separation of electron-rich and electron-poor microenvironments creates a strong thermodynamic driving force for EET between microbial populations, potentially enabling electrically mediated syntrophy (15–17) and contributing to redox homeostasis (18, 19) within the gut ecosystem.

EET has been documented in diverse microorganisms inhabiting soil and sediments (20–22), oral environments (23, 24), and mammalian guts (25, 26) and can proceed through multiple mechanistic routes. These mechanisms have been most extensively characterized in gram-negative genera such as *Shewanella*, *Geobacter*, and *Desulfovibrio*, where EET involves self-secreted soluble redox mediators (22), outer-membrane cytochromes (20, 27, 28), conductive nanowires (29–31), or membrane-associated conductive nanoparticles (32). In contrast, EET mechanisms in gram-positive bacteria, including many cellulolytic lineages such as members of the class *Clostridia*, remain much less explored. Redox-active lipoproteins and exogenous flavins have been proposed to mediate EET in the pathogenic bacterium *Listeria monocytogenes* (33).

We evaluated the EET capability and mechanism of the termite gut microbes by directly measuring anodic current production from a cellulolytic termite gut isolate, *Ruminiclostridium cellobioparum* subsp. *termitidis* CT1112, as well as from the gut microbiome of *Reticulitermes speratus*, a subterranean termite species prevalent in Japan. These measurements link cellulose degradation to extracellular redox fluxes at both the single-strain and community levels.

## RESULTS

To determine whether cellulose-driven electron transfer could be sustained by a single cellulolytic bacterial species, we examined *R. cellobioparum* subsp. *termitidis* CT1112, a gram-positive, spore-forming member of the phylum Bacillota isolated from the hindgut of *Nasutitermes lujae* (34, 35). In electrochemical reactors supplied with microcrystalline cellulose powder, sodium carboxymethyl cellulose (CMC), or cellobiose as the electron donor and a 0.4 V (versus standard hydrogen electrode)-poised electrode as the electron acceptor, sterile electrolytes produced only background currents (<0.002 µA cm⁻^2^; Fig. 1a). Anodic currents increased rapidly upon introduction of CT1112 cells, which were harvested during the exponential growth phase using centrifugation. Cellobiose supported the highest current density, followed by CMC and cellulose. In contrast, autoclave-inactivated CT1112 cells produced no measurable current in the presence of cellobiose. Consistently, cyclic voltammetry performed after current measurement revealed distinct pairs of redox peaks with midpoint potentials of 67, 13, and −30 mV in current-producing reactors with cellobiose, CMC, and cellulose, respectively (Fig. 1b), indicating the presence of redox-active components associated with the metabolism of cellulose and its derivatives. The substrate-dependent shifts in midpoint potential suggest that different carbon sources may influence the production, abundance, or redox state of extracellular redox-active components. Moreover, with cellobiose selected as a representative electron donor, we further evaluated the effect of EET on intracellular redox balance. The cellular NAD^+^/NADH ratio increased from approximately 1.6 under open-circuit conditions, a value comparable to those reported for several bacterial strains under anaerobic fermentative conditions (36, 37), to approximately 3.2 under EET conditions (Fig. 1c; Fig. S1). An elevated NAD^+^/NADH ratio under EET conditions has also been reported for *Lactiplantibacillus plantarum* (38). These results suggest that a portion of electrons derived from cellobiose metabolism was redirected to EET, thereby shifting the intracellular redox balance toward a more oxidized state. Collectively, these results demonstrate that cellulose-derived substrates can support EET in a single cellulolytic bacterial species.

**Fig 2:**
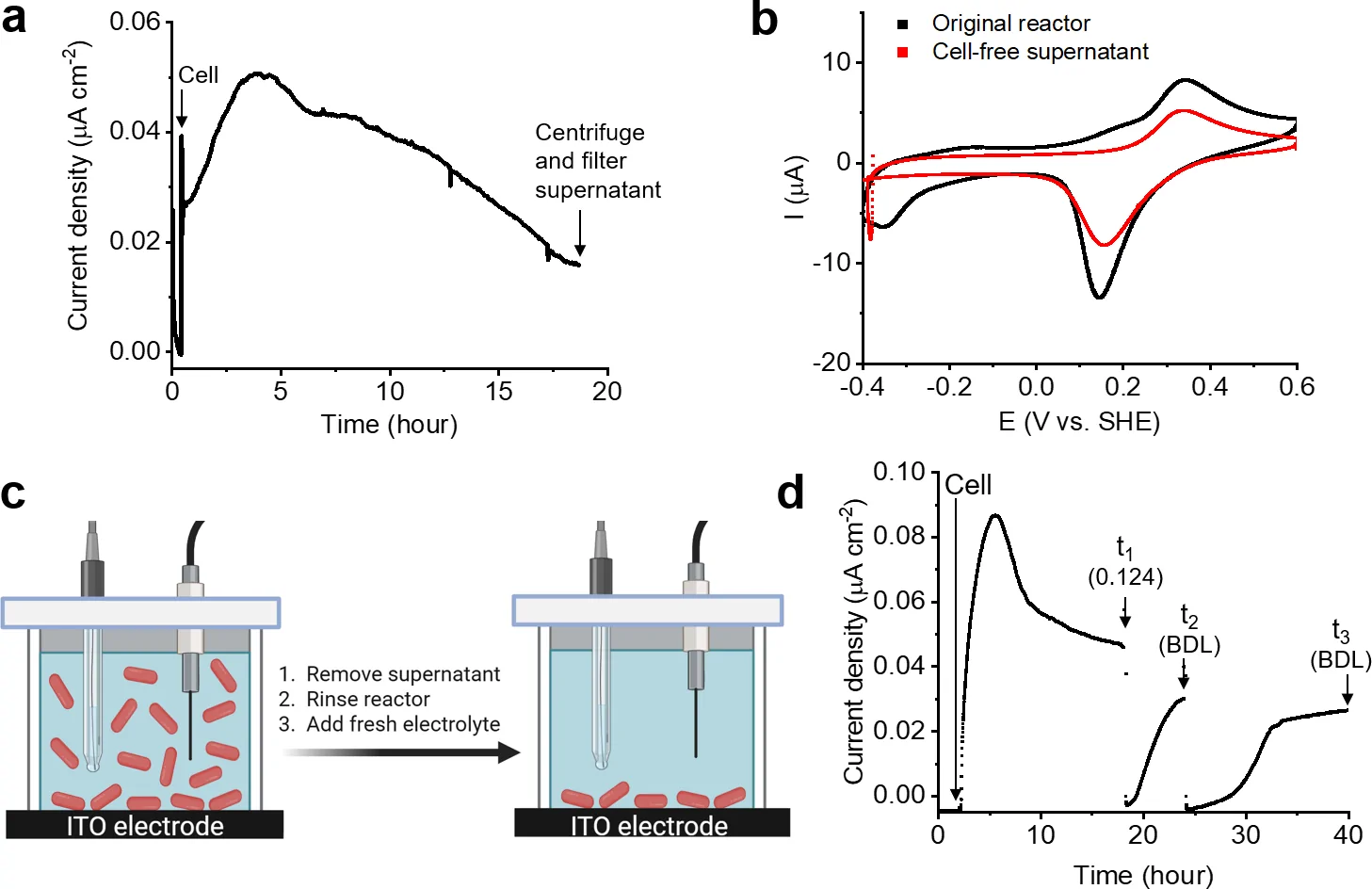
Involvement of self-secreted redox mediators in the extracellular electron transfer (EET) process of CT1112 cells. (**a**) Representative current production by CT1112 cells with cellobiose as the electron donor. A cyclic voltammogram was recorded in the original reactor at 19 h. The electrolyte supernatant was then collected, centrifuged, and filtered to obtain a cell-free supernatant. (**b**) Comparison of cyclic voltammograms recorded at 19 h for the original reactor and the cell-free supernatant. (**c**) Schematic of the supernatant exchange experiment. (**d**) Current production with electrolyte/supernatant exchanges. Supernatants were exchanged at times *t*_1_ and *t*_2_. Brackets indicate the cell density (OD_600nm_) of the planktonic phase measured at *t*_1_, *t*_2_, and *t*_3_. BDL, below detection limit.

The produced redox-active signals were most likely assignable to self-secreted soluble redox-active mediators. The redox peaks at identical potentials were detected in both the original reactor and the cell-free reactor supernatant (Fig. 2a and b). However, the lower peak intensity in the cell-free supernatant suggests that a substantial fraction was either cell-associated or lost during sample processing, such as through filter adsorption. Replacing the electrolyte containing soluble metabolites and planktonic cells in the electrolyte supernatant exchange experiment (20–22) (Fig. 2c) with fresh sterile electrolyte after CT1112 had maintained stable anodic current production for more than 15 h (Fig. 2d) resulted in an immediate and marked drop in current. The current then recovered to approximately 80% of the pre-exchange level over several hours, even though planktonic cells remained below the detection limit (Fig. 2d). Repeating the supernatant exchange at 25 h yielded a similar response, demonstrating that the current generation was primarily mediated by electrode-attached cells but required soluble, self-secreted redox shuttles.

**Fig 3:**
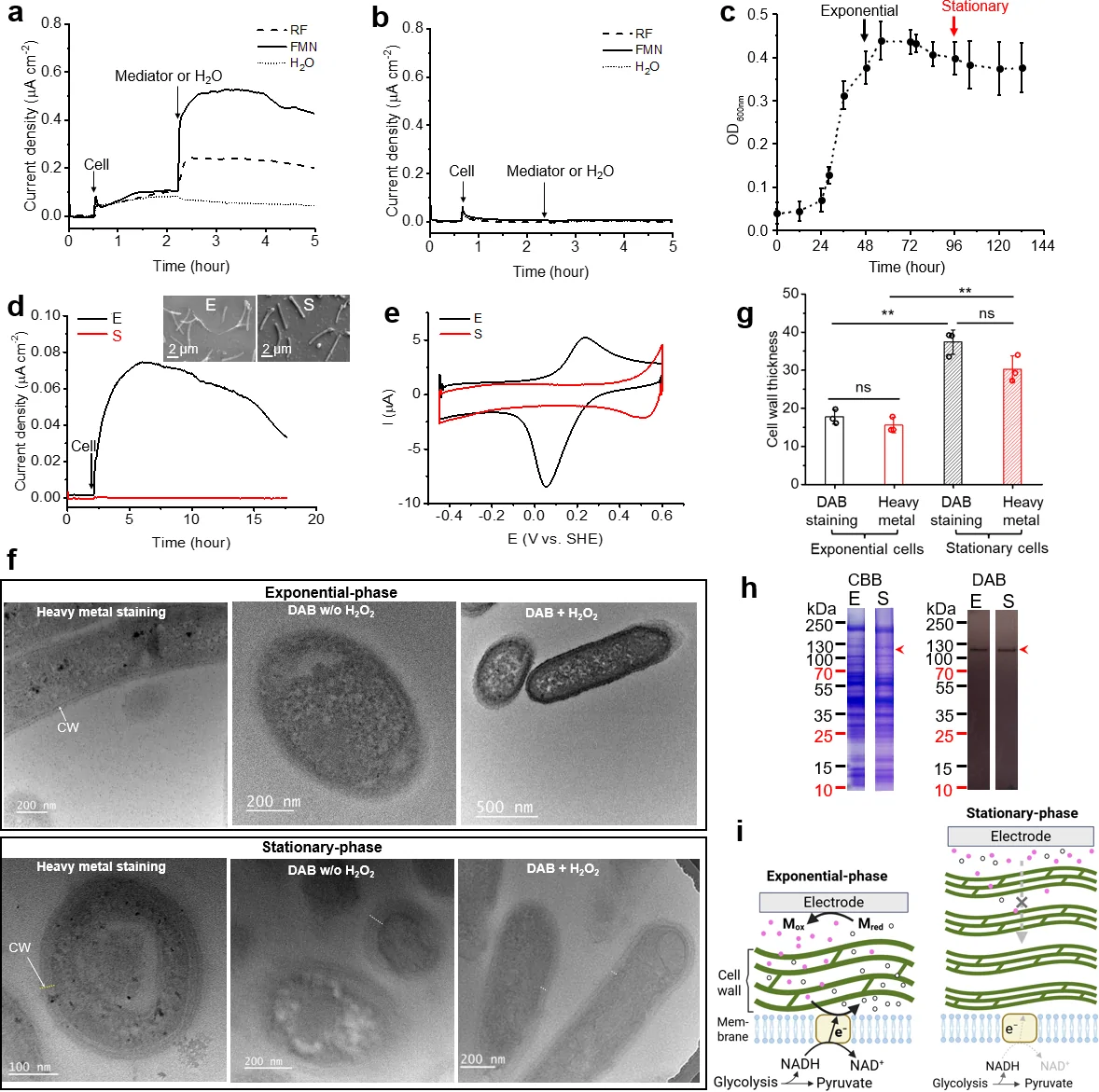
Cell wall permeability to redox shuttles limits extracellular electron transfer (EET) in CT1112 cells. (**a and b**) Effects of 20 µM riboflavin (RF) and flavin mononucleotide (FMN) on current production by cells precultured to the exponential (E) and stationary (S) phases. (**c**) Cell growth curve on cellobiose. Arrows indicate when E- and S-phase cells were collected. (**d**) Current production by E- and S-phase cells with cellobiose as the electron donor. Inset: scanning electron microscopy (SEM) image of the electrode surface. (**e**) Cyclic voltammograms recorded after current measurement. (**f**) Transmission electron microscopy (TEM) images of ultrathin cell sections. Heavy-metal staining was performed on ultrathin sections, whereas 3,3′-diaminobenzidine (DAB) staining was performed on intact cells before sectioning. Dotted lines indicate the thickened cell wall. (**g**) Quantification of cell wall thickness based on TEM measurements of three individual cells. *P* < 0.01 (two-sided *t*-test); ns, not significant. (**h**) Coomassie Brilliant Blue (CBB) and DAB staining of extracted cytoplasmic membrane fractions, showing a DAB-positive band in both phases, in which pyruvate:ferredoxin (flavodoxin) oxidoreductase (128.7 kDa; *CTER_3589*) was detected. (**i**) Schematic illustration of the effect of cell wall permeability on EET.

Because flavins are among the best-characterized soluble mediators of microbial EET, including in gram-positive bacteria (33), we next used riboflavin (RF) and flavin mononucleotide (FMN) as representative exogenous mediators to evaluate whether flavins could enhance current production by CT1112. Upon the addition of RF (*E°′* = −210 mV) or FMN (*E°′* = −220 mV), the current production increased within 2 min; in contrast, the water addition yielded no such increase (Fig. 3a). These results further support the idea that common soluble redox mediators can facilitate EET in CT1112. Nonetheless, given that the redox potentials of the redox-active species detected with CT1112 cells and the cell-free supernatant differed substantially from those of RF and FMN, the redox mediator produced by CT1112 is likely distinct from these flavins.

While CT1112 cells precultured to the exponential phase produced current, cells precultured to the stationary phase were deficient in both current production and redox signal generation (Fig. 3b through e). This deficiency could not be restored by adding exogenous RF (376 Da) or FMN (456 Da), both of which enhanced EET in exponential-phase cells (Fig. 3a and b). Additionally, we confirmed that stationary-phase cells remained viable (Fig. S2) and attached to the electrode at levels comparable to those of exponential-phase cells (Fig. 3d inset).

Given that precultivation into the stationary phase may induce cell wall thickening and remodeling, including reduced permeability (39–42), the EET kinetics may be suppressed. Transmission electron microscopy (TEM) revealed that stationary-phase cells exhibited a markedly thickened, densely packed, multilayered architecture, contrasting the relatively thin, single-layered cell wall of exponential-phase cells (Fig. 3f and g; Fig. S3 and S4). Considering that heavy metals stain the negatively charged peptidoglycan matrix and wall-associated teichoic acids, the multilayered structures may represent peptidoglycan-enriched bundles, similar to observations reported in another study (43), although their exact composition remains unresolved. The thickened cell wall had reduced permeability to small redox molecules, as demonstrated using diaminobenzidine (DAB) staining of intact cells to assess whether DAB (214 Da) could diffuse across the cell wall to reach the DAB-reactive protein localized to the cytoplasmic membrane (Fig. 3h; Fig. S5). Staining intact cells with DAB resulted in electron-dense deposition at the membrane in exponential-phase cells, but not in stationary-phase cells (Fig. 3f; Fig. S6 and S7), indicating that DAB did not diffuse through the stationary-phase cell wall. These results further support the idea that diffusion of redox mediators across the cell wall is a critical process for EET in CT1112 (Fig. 3i).

**Fig 4:**
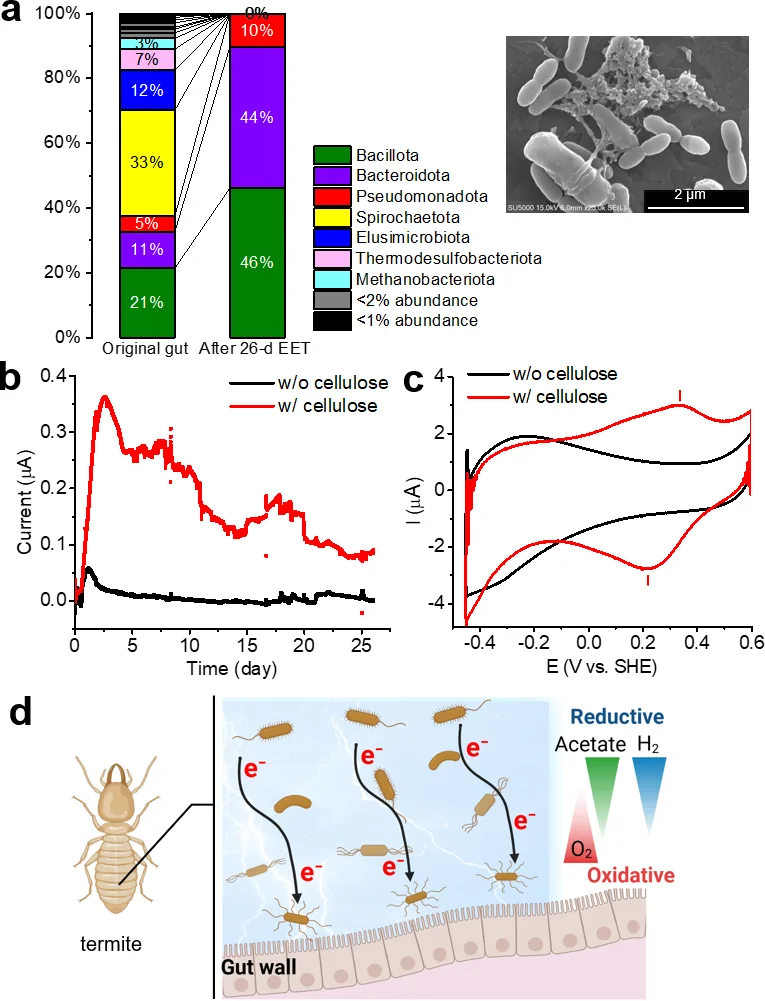
Cellulose-coupled extracellular electron transfer (EET) in the gut microbiome of the termite *Reticulitermes speratus*. (**a**) Phylum-level composition (top 7 phyla colored) of the termite gut microbiome before and after 26 days of EET operation. Gray and black boxes denote the remaining phyla with low relative abundance (<2% and <1%, respectively). (**b**) Anodic current production by termite gut microbes with or without the addition of microcrystalline cellulose powder. (**c**) Cyclic voltammogram recorded on day 17. Red bars indicate a pair of redox peaks. (**d**) Schematic illustration of steep redox gradients in the termite gut, providing a strong driving force for EET.

Finally, we analyzed the EET capability using the gut microbiome of *Reticulitermes speratus*, a termite species prevalent in Japan, to test whether cellulose-fueled EET is a broader community-level trait of termite gut microbes. The gut microbiome comprised a complex assemblage of bacterial and archaeal phyla, including Spirochaetota (33%), Bacillota (21%), Elusimicrobiota (12%), Bacteroidota (11%), Thermodesulfobacteriota (7%), Pseudomonadota (5%), and the archaeal phylum Methanobacteriota (3%), together with other low-abundance lineages (Fig. 4a), consistent with previously reported termite gut communities (8, 44, 45). This community was introduced into an electrochemical reactor equipped with an electrode poised at 0.4 V, with microcrystalline cellulose powder supplied as the electron donor, to evaluate whether it could couple cellulose degradation to EET; an identical reactor lacking cellulose served as the negative control. Sterile electrolytes, with or without cellulose, generated negligible current. In contrast, the cellulose-containing reactor exhibited a rapid increase in anodic current, exceeding 0.36 μA within 3 days upon microbiome addition (Fig. 4b). Although the current gradually declined thereafter, it remained above 0.1 μA after 26 days of operation. No sustained increase in current was observed in reactors lacking cellulose. Cyclic voltammetry performed on day 17 revealed a distinct pair of redox peaks with a midpoint potential of 0.27 V exclusively in the cellulose-amended system (Fig. 4c). This indicated the presence of redox-active components associated with cellulose metabolism. Collectively, these results demonstrate that the termite gut microbiome can also transfer electrons to an external anode during cellulose degradation.

Notably, the gut microbial community exhibited a pronounced shift in taxonomic composition after 26 days of producing current, with Bacillota (46%), Bacteroidota (44%), and Pseudomonadota (10%) collectively dominating the consortium (Fig. 4a). Bacillota largely comprise gram-positive fermentative bacteria (46), whereas Bacteroidota include gram-negative carbohydrate-degrading taxa (47), and Pseudomonadota contain lineages previously associated with EET (18). This compositional shift reflects changes in the relative abundance of termite gut microbial populations under cellulose-driven electrochemical conditions. Furthermore, scanning electron microscopy (SEM) revealed direct attachment of cells to the electrode surface, as well as filamentous structures ranging from several tens of nanometers to a few micrometers in length (Fig. 4a inset panel; Fig. S8), indicating both cell-electrode and cell-cell interactions.

## DISCUSSION

In this study, we demonstrated cellulose-fueled EET in both a gut-derived cellulolytic bacterium and the termite gut microbiome, suggesting that extracellular redox fluxes driven by cellulosic matter degradation may be widespread in termite-associated communities and that EET may play a broader role in biogeochemical carbon cycling. Mechanistic analysis of the pure cellulolytic strain CT1112 revealed that the generation of current required self-secreted soluble redox components, whose electron-shuttling processes critically depended on cell wall permeability, a phenomenon not previously reported. Contrary to the traditional view that the cell walls of gram-positive bacteria are permeable to macromolecules (42), our results indicate that diffusion limitations can arise even for molecules as small as a few hundred Da when the peptidoglycan architecture and physicochemical properties are substantially altered (48, 49). The rapid current increase upon introduction of centrifuged and resuspended CT1112 cells suggests that the contributor to EET was most likely self-secreted redox mediators, because other potential factors, such as membrane vesicles or membrane fragments, were not observed on the electrode surface or cells in SEM and TEM analyses, respectively, either before or after current measurements.

We further demonstrated cellulose-fueled EET in the termite gut microbiome. Termite diets include redox-active soil components such as iron(III) oxides and humic substances (14), and the reduction of iron(III) hydroxides to iron(II) has been reported in termite guts (50). Therefore, EET coupled to insoluble external electron acceptors may represent an underappreciated mechanism for redox balancing within the gut environment (Fig. 4d).

Although redox-active proteins associated with the cell wall could not be predicted from the genome sequence (SI Table S1 ) or detected in the extracted cell wall using the DAB staining method (Fig. S9), completely ruling out a direct EET route will require further experimental validation, such as proteomic and electrochemical characterization of isolated cell wall fractions. Our NAD^+^/NADH measurements suggest that EET functions as a redox-balancing route to dissipate excess reducing equivalents in CT1112. Based on genome analysis, NAD^+^ regeneration in CT1112 may be supported by two systems (Fig. S10 and Table S2). First, an electron-bifurcating [FeFe] hydrogenase likely couples the oxidation of NADH and reduced ferredoxin, produced by pyruvate:ferredoxin (flavodoxin) oxidoreductase, to proton reduction. Second, the genome encodes a partial complex I-like module comprising NuoF, NuoB/C, and the membrane-associated NuoK/H/L but lacks genes for ubiquinone or menaquinone biosynthesis. This raises the possibility that electrons downstream of NuoF and NuoB/C are directed to extracellular carriers. Consistent with this idea, NADH dehydrogenase components in *Pseudomonas aeruginosa* have been implicated in the reduction of redox mediators such as phenazine (51). Targeted deletion of Nuo components will be required to determine whether and how electrons from the NuoF-NuoB/C module are transferred to soluble redox mediators.

This study also highlighted termite-derived microbial consortia as promising platforms for the development of cellulose-based bioelectrochemical applications, such as cellulolytic microbial fuel cells (MFCs) (6, 7). The current densities observed in this study were several hundred-fold lower than the peak current densities reported for cellulose- or CMC-fueled MFCs (100–800 mA m^−2^, equivalent to 10–80 μA cm^−2^), suggesting that CT1112 and the gut microbiome have intrinsically low EET activity under the tested conditions. Additionally, diffusion limitations of soluble redox mediators within the cell-associated extracellular matrix and between cells and the electrode may also have contributed to the low current densities. However, future improvements in the electrode-microbe interface (52) and optimization of operating conditions, including cell density and mediator addition (53), may substantially enhance the performance of termite gut-derived microbial systems.

Given that cellulose constitutes one of the largest reservoirs of fixed organic carbon in terrestrial ecosystems (1) and that termites are among its most efficient degraders (1, 54), our findings extend the known occurrence of EET from systems utilizing soluble substrates to the degradation of the planet’s dominant structural carbon polymer. In this context, extracellular redox processes may contribute directly to carbon flow during lignocellulose degradation in natural anaerobic microsystems. While EET in termite guts could potentially influence electron partitioning among fermentation, mineral reduction, and other microbial processes, our findings also raise the possibility that the interspecies electron transfer process contributes to metabolic cooperation within termite gut communities. In future, uncovering EET and extracellular electron uptake pathways across diverse termite microbiomes may improve our understanding of how microbial consortia govern carbon turnover and redox balancing in lignocellulose-rich anaerobic environments.

## MATERIALS AND METHODS

### Cultivation of *R. cellobioparum* subsp. *termitidis* CT1112

Strain CT1112 (DSM No. 5398) was obtained from the Deutsche Sammlung von Mikroorganismen und Zellkulturen (DSMZ) and cultivated in glass vials containing 60 mL of growth medium (https://www.dsmz.de/microorganisms/medium/pdf/DSMZ_Medium539.pdf, accessed 22 April 2026) containing 5 g/L cellobiose as the electron donor. Cultures were incubated at 37°C under an N_2_/CO_2_ (80:20, vol/vol) headspace without agitation. Cell growth was monitored by measuring optical density at 600 nm using a Multiskan SkyHigh spectrophotometer (Eppendorf, Hamburg, Germany).

### Electrochemical analyses

Electrochemical analyses were conducted in single-chamber, three-electrode reactors using a potentiostat VMP3 (BioLogic, Seyssinet-Pariset, France). The reactors were maintained at 37°C in an anaerobic chamber (Coy Laboratory Products, Grass Lake, MI, USA) under a 100% N_2_ atmosphere. An indium-tin-doped oxide (ITO) electrode (3.14 cm^2^, sheet resistance 5 Ω/sq) was used as the working electrode and placed at the bottom. An Ag/AgCl (saturated KCl) electrode and a platinum wire were used as the reference and counter electrodes, respectively. The electrolyte composition was as follows (per liter): KH_2_PO_4_ 0.2 g, NH_4_Cl 0.3 g, NaCl 1.0 g, MgCl_2_⋅6H_2_O 0.4 g, KCl 0.5 g, CaCl_2_⋅2H_2_O 0.15 g, yeast extract 0.5 g, trace element solution SL-10 1 mL, Na_2_CO_3_ 1.0 g, and 25 mM HEPES [4-(2-hydroxyethyl)−1-piperazineethanesulfonic acid] buffer (pH 7.2). Microcrystalline cellulose powder, CMC (Product No. 419273; Sigma-Aldrich, St. Louis, MO, USA), and cellobiose were supplemented to the electrolyte to a final concentration of 5 g/L and tested as the electron donor. The electrolyte was purged with N_2_ for 15 min, autoclaved (121°C, 15 min), and then purged again with N_2_ for an additional 15 min before use.

For chronoamperometric measurements of anodic current production, the ITO electrode was poised at 0.4 V (vs SHE) to serve as an external electron acceptor. Exponential- and stationary-phase cells were harvested from 1- and 4-day-old cultures, respectively, by centrifugation (7,800 × *g* for 10 min) in the anaerobic chamber under a 100% nitrogen atmosphere. Cell pellets were resuspended in 0.5 mL of anoxic electrolyte and inoculated into reactors containing 4.5 mL of electrolyte, resulting in a final working volume of 5 mL and an initial OD_600nm_ of 0.1. Cyclic voltammetry was recorded over an electrode potential range of −0.45 to 0.6 V at a scan rate of 20 mV/s.

For electrolyte exchange experiments, chronoamperometric measurements were paused, and the electrolyte, including planktonic cells, was completely removed using a syringe. The reactor interior, including the ITO electrode, was rinsed with 4 mL of fresh electrolyte, and the rinse solution was discarded. Subsequently, 5 mL of fresh electrolyte was added, and chronoamperometric measurements were resumed. For measurement of OD_600nm_ of planktonic cells, 0.5 mL of electrolyte was withdrawn from the midpoint of the reactor using a syringe and measured immediately. For measurement of redox-active species in the cell-free supernatant, the reactor contents were first centrifuged at 7,800 × *g* for 10 min to remove cells. The resulting supernatant was then passed twice through sterile 0.22 µm pore-size filters. The filtrate was subsequently transferred to a fresh electrochemical reactor for cyclic voltammetry analysis.

All electrochemical measurements involving CT1112 cells were independently replicated at least three times, and representative data are shown in the figures.

### NAD^+^/NADH ratio measurement

After incubation of CT1112 cells for 8 h in electrochemical reactors equipped with ITO electrodes poised at 0.4 V (EET condition) or maintained under open-circuit conditions, cells in the reactors, including those attached to the ITO electrodes, were collected and disrupted inside a COY anaerobic chamber. Specifically, the culture supernatants were transferred to centrifuge tubes, and electrode-attached cells were recovered by scraping with a pipette tip. The collected cells were pelleted by centrifugation at 7,800 × *g* for 10 min at room temperature, resuspended in 150 µL of N_2_-purged anoxic phosphate-buffered saline (PBS; 140 mM NaCl, 2.7 mM KCl, 10 mM phosphate buffer, pH 7.4), and disrupted using EZ-Beads at 4°C (Promega, Madison, WI, USA) according to the manufacturer’s instructions. Following bead disruption, the lysate-bead mixture was centrifuged at 15,100 × *g* for 3 min at 4°C, and 50 µL of the resulting supernatant was collected. The supernatant was then removed from the anaerobic chamber and immediately used for NAD^+^/NADH ratio measurement with the NAD/NADH-Glo Assay Kit (Promega, Madison, WI, USA) according to the manufacturer’s instructions.

### Scanning electron microscopy

After electrochemical measurements, ITO electrodes with attached cells were removed from the reactors and immediately fixed by immersion in 2.5% (vol/vol) glutaraldehyde in phosphate buffer (PB; 0.1 M, pH 7.0) for 1 h. The electrodes were then immersed three times in fresh PB for 8 min each, followed by dehydration through a graded ethanol series (25%, 50%, and 75% [vol/vol] in ultrapure water, and 100% ethanol), with 8 min per step. Ethanol was subsequently exchanged three times with *tert-*butanol, and the samples were freeze-dried under vacuum. Dried samples were coated with evaporated platinum using a Quick Coater SC-701 (Sanyu Denshi K.K., Nagoya, Japan) and examined using SU8000 (Hitachi, Tokyo, Japan).

### Extraction of cell wall and cytoplasmic membrane

CT1112 cells were cultivated in 3 L of growth medium for one or 4 days, and exponential- and stationary-phase cells were harvested by centrifugation at 7,800 × *g* for 15 min. Approximately 3 g (wet weight) of cell pellet was obtained, immediately frozen in liquid nitrogen, and stored at −80°C overnight. Frozen pellets were thawed at room temperature and resuspended in 25 mL of lysis buffer (50 mM Tris-HCl, pH 7.5; 1 mM EDTA; 1 mg/mL lysozyme; and 1 × Halt Protease Inhibitor Cocktail [Product No. 78430, Thermo Fisher Scientific, Waltham, MA, USA]), followed by incubation at 37°C for 30 min on a tube rotator.

The suspension was then kept at 4°C and further disrupted using a probe sonicator/homogenizer Branson Sonifier 450 (Branson Ultrasonics, Danbury, USA) at 30% amplitude for 24 cycles (10 s on, 30 s off). Unbroken cells were removed by centrifugation at 8,000 × *g* for 8 min at 4°C, repeated three times, until no visible pellet remained. The resulting supernatant (turbid) was used for fractionation of crude cell wall and membrane components.

Crude cell wall was collected by centrifugation of the clarified lysate at 20,000 × *g* for 20 min at 4°C. The pellet was resuspended in 5 mL of 50 mM Tris-HCl (pH 7.5) containing 1% (vol/vol) Triton X-100 and incubated for 25 min at room temperature with gentle agitation (500 rpm; MixMate, Eppendorf, Hamburg, Germany) to solubilize cytoplasmic membrane components. The Triton X-100 extraction was repeated once, and the cell wall fraction was pelleted again by centrifugation at 20,000 × *g* for 20 min. Residual Triton X-100 was removed by washing the cell wall pellet twice with 50 mM Tris-HCl (pH 7.5) (resuspension by pipetting followed by centrifugation). The final cell wall pellet was resuspended in 1 mL of 50 mM Tris-HCl (pH 7.5) and used for electrophoresis.

Crude cytoplasmic membrane was pelleted from the clarified lysate by ultracentrifugation at 180,000 × *g* for 2 h. The pellet was resuspended in 50 mM Tris-HCl (pH 8.0) and layered onto a sucrose density gradient for further purification. Membrane fractions (lower density) were separated from higher-density cell wall material by ultracentrifugation at 82,000 × *g* for 17 h. After centrifugation, 12 fractions for the stationary-phase sample and 13 fractions for the exponential-phase sample were collected sequentially from the top to the bottom of the gradient; membrane fractions were recovered from the upper layers, whereas cell wall-associated material was enriched in deeper layers.

### Protein analysis of cell wall and cytoplasmic membrane fractions

Purified cell wall and cytoplasmic membrane fractions were analyzed by sodium dodecyl sulfate-polyacrylamide gel electrophoresis. Following electrophoresis, gels were stained with EzStain Aqua (ATTO, Tokyo, Japan) according to the manufacturer’s instructions. For detection of redox-active proteins, gels were first rinsed with distilled water and then incubated in a 0.22 µm filtered DAB staining solution for 1 h with gentle rocking. The DAB solution was prepared by dissolving 0.04 g DAB in 1 mL of 1 M HCl, followed by dilution with 29 mL of 50 mM Tris-HCl buffer (pH 8.0). Hydrogen peroxide was then added to a final concentration of 0.02% (vol/vol) (20 µL of 30% H_2_O_2_), and gels were incubated for an additional 30 min. Gels were then washed five times with distilled water (5 min each) and subsequently immersed in 10 mL of 0.04% (wt/vol) OsO_4_ to enhance signal development. Distinct protein bands became visible within 3 min.

### Prediction of putative cell wall- and cytoplasmic membrane-localized redox proteins from the CT1112 genome sequence

The amino acid sequences encoded in the CT1112 genome were analyzed using PSORTb (55) to predict subcellular localization. Proteins with a final prediction of cell wall or cytoplasmic membrane were extracted, and the corresponding gene loci were arranged in genomic order. All 64 proteins predicted to localize to the cell wall were annotated as proteins with known functions and did not include any obvious EET-associated electron-transfer proteins. In contrast, 1,491 proteins were predicted to localize to the cytoplasmic membrane. Among these, proteins with known redox functions, including NADH oxidoreductases, hydrogenases, and complex I-like subunits, were selected for further examination of their potential roles in dissipating excess reducing equivalents through EET.

### Transmission electron microscopy

Exponential- and stationary-phase CT1112 cells were harvested from 15 mL of 1- and 4-day-old liquid cultures, respectively, by centrifugation (7,800 × *g*, 10 min) inside an anaerobic chamber. Cell pellets were resuspended and fixed in 4% paraformaldehyde and 2% glutaraldehyde at 4°C for 17 h. Fixed cells were washed three times with PBS and pelleted by centrifugation (7,800 × *g*, 10 min).

For heavy-metal staining, paraformaldehyde- and glutaraldehyde-fixed cells were postfixed with 1% OsO_4_ at 4°C for 2 h and then washed three times with preboiled and cooled 25 mM HEPES buffer (pH 7.4) by centrifugation at 8,000 × *g* for 8 min. The cells were dehydrated through a graded ethanol series (25%, 50%, and 75% [vol/vol] in ultrapure water, followed by two changes of 100% ethanol; 15 min per step) and infiltrated with a 1:1 (vol/vol) mixture of ethanol and LR White for 1 h at room temperature with gentle rotation. This was followed by infiltration with 100% LR White for 1 h at room temperature with gentle rotation. Fresh LR White was then added, and samples were polymerized at 60°C for 48 h. Ultrathin sections (80 nm) were cut using an ultramicrotome EM UC7 (Leica Microsystems GmbH, Wetzlar, Germany), mounted on copper microgrids. The microgrids were stained with heavy metals using EM stainer (Nisshin EM, Tokyo, Japan) for 30 min at room temperature, rinsed eight times with preboiled, cooled distilled water, air-dried for 5 min, and counterstained with lead citrate for 3 min. The grids were then rapidly rinsed with preboiled, cooled distilled water, air-dried for 5 min, and subjected to a second round of lead citrate staining to enhance contrast. Sections were examined using a transmission electron microscope JEM-1400 (JEOL Ltd., Tokyo, Japan) operated at 80 kV.

For redox staining, cell pellets were treated by a 3,3′-DAB-staining method (15, 30, 56). Briefly, the pellets were incubated in 1.5 mL of DAB solution (5.7 mg DAB dissolved in 0.1 mL of 1 M HCl; diluted with 3.7 mL of 50 mM Tris buffer, pH 8.0; and filtered through a 0.22 μm filter), either with 1 μL of H_2_O_2_ (positive DAB staining) or without H_2_O_2_ (negative DAB staining), for 2 h at room temperature in the dark with gentle rotation. The cells were washed five times with 100 mM HEPES buffer (pH 7.8) by centrifugation at 8,000 × *g* for 8 min. The cells were subsequently stained with 1% OsO_4_ by incubation on ice for 1 h with gentle rocking, followed by five washes with the same HEPES buffer. Dehydration, embedding in LR White resin, thin sectioning, and TEM imaging were performed as described above.

### Sampling of the gut microbes from *Reticulitermes speratus*

*Reticulitermes speratus* individuals were collected from Enjugahama Pine Forest, Wakayama Prefecture, Japan (33.893885°N, 135.126902°E) and transported to the laboratory at 4°C. Five live termites were transferred into an anaerobic chamber under a 100% nitrogen atmosphere and immediately degutted using sterilized tweezers, and the isolated guts were pooled and thoroughly homogenized using sterile pipette tips to release the gut microbiome into 1 mL of sterile, anoxic saline solution (0.85% NaCl). The resulting microbial suspension was divided into two aliquots: one aliquot was pelleted by centrifugation at 10,000 × *g* for 10 min and stored at −80°C for microbial taxonomic analysis, while the other was used for electrochemical analysis.

### Gut microbiome DNA extraction and sequencing

While the original gut microbes released from termite guts were stored at −80°C, 0.5 mL of the gut microbial suspension from electrochemical reactors operated at +0.4 V for 26 days was collected, centrifuged at 10,000 × *g* for 10 min, and stored at −80°C. The cell pellets were lysed in Lysis Solution F (Nippon Gene, Tokyo, Japan), homogenized at 1,500 rpm for 2 min (Shake Master NEO BMS, Tokyo, Japan), incubated at 65°C for 10 min, and centrifuged at 12,000 × *g* for 2 min. DNA was purified from the supernatant using the Lab-Aid 824s DNA Extraction Kit (ZEESAN, Xiamen, China).

Amplicon libraries targeting the 16S rRNA V4 region were prepared using a two-step tailed PCR approach with the 515f/806rb primer set and sequenced on a NextSeq 1000 system (Illumina, San Diego, CA, USA) using the NextSeq 1000/2000 P1 XLEAP-SBS Reagent Kit (600 cycles) in 2 × 300 bp mode. Reads with perfect primer matches were retained using *fastx_barcode_splitter* and trimmed with *fastx_trimmer*. Low-quality reads (*Q* < 20) and reads <130 bp were removed using *sickle* (v1.33). Paired-end reads were merged using *FLASH* (v1.2.11). Denoising and chimera removal were performed with DADA2 in QIIME2 (v2025.7) to generate amplicon sequence variants (ASVs). No additional singleton filtering was applied after ASV inference. Taxonomic assignment was conducted using the QIIME2 *feature-classifier* against the SILVA database (v138.2).

## Data Availability

The 16S rRNA gene amplicon sequencing data have been deposited in the NCBI Sequence Read Archive (SRA) under BioProject number PRJNA1482034.

## SUPPLEMENTAL MATERIAL

Supplemental material aem.01272-26-s0001.docx Fig. S1 to S10; Tables S1 and S2.

ASM does not own the copyrights to Supplemental Material that may be linked to, or accessed through, an article. The authors have granted ASM a non-exclusive, world-wide license to publish the Supplemental Material files. Please contact the corresponding author directly for reuse.
