# Supplementary material for: Cellulose-fueled extracellular electron transfer in termite gut microbes
Source: Appl Environ Microbiol. 2026 Aug 27;92(9):e01272-26. doi: 10.1128/aem.01272-26 (PMC13599477; doi:10.1128/aem.01272-26)
Supplement: Supplementary file 1 [file aem.01272-26-s0001.docx]

**Supporting Information**

**Cellulose-Fueled Extracellular Electron Transfer in Termite Gut Microbes**

Xiao Deng^a,#^ and Akihiro Okamoto^a,b,c,d^

^a^Research Center for Macromolecules and Biomaterials, National Institute for Materials Science, Namiki 1-1, Tsukuba, Ibaraki 305-0044, Japan

^b^Graduate School of Chemical Sciences and Engineering, Hokkaido University, Kita 13, Nishi 8, Kita-ku, Sapporo 060-8628, Japan

^c^Graduate School of Science and Technology, University of Tsukuba, 1-1-1 Tennodai, Tsukuba, 305-8577, Japan

^d^Research Center for Autonomous Systems Materialogy, Institute of Science Tokyo, 4259 Nagatsuta-cho, Midori-ku, Yokohama, Kanagawa 226-8501, Japan

^#^Address correspondence to Xiao Deng: deng.xiao@nims.go.jp

This file includes

10 Supporting Figures

2 Supporting Tables

Figure S1. Anodic current production by CT1112 cells on a +0.4 V (vs SHE)-poised electrode over 8 h prior to NAD^+^/NADH measurement.

Figure S2. Growth curve of CT1112 cells in medium containing cellobiose as the electron donor, using exponential- or stationary-phase cells as the inoculum at a concentration of 3%.

Figure S3. Transmission Electron Microscopy (TEM) images of ultrathin sections of exponential-phase CT1112 cells used for cell wall thickness measurements. Sections were contrasted using heavy-metal staining (EM stainer, Nisshin EM, Japan) after being mounted on TEM grids. Line profiles across the cell wall are shown; the y-axis represents electron transmission intensity.

Figure S4. Transmission Electron Microscopy (TEM) images of ultrathin sections of stationary-phase CT1112 cells used for cell wall thickness measurements. Sections were contrasted using heavy-metal staining (EM stainer, Nisshin EM, Japan) after being mounted on TEM grids. Line profiles across the cell wall are shown; the y-axis represents electron transmission intensity.

Figure S5. Sodium dodecyl sulfate-polyacrylamide gel electrophoresis (SDS-PAGE) analysis of membrane fractions isolated from exponential- and stationary-phase CT1112 cells. (a) Coomassie Brilliant Blue (CBB)-stained gel showing total proteins. (b) 3,3′-diaminobenzidine (DAB)-stained gel for detection of redox-active proteins. Lanes 1–13 and lanes 1–12 correspond to fractions sequentially collected from the top to the bottom of a sucrose density gradient following ultracentrifugation.

Figure S6. Transmission Electron Microscopy (TEM) images of ultrathin sections of 3,3′-diaminobenzidine (DAB)-stained exponential-phase CT1112 cells. Positive DAB staining was performed on intact cells prior to sectioning. Line profiles across the cell wall are shown; the y-axis represents electron transmission intensity.

Figure S7. Transmission Electron Microscopy (TEM) images of ultrathin sections of 3,3′-diaminobenzidine (DAB)-stained stationary-phase CT1112 cells. Positive DAB staining was performed on intact cells prior to sectioning. Line profiles across the cell wall are shown; the y-axis represents electron transmission intensity.

Figure S8. Scanning electron microscopy images of termite gut microbes attached to the indium-tin-doped oxide (ITO) electrode surface after 26 days of electrochemical operation at 0.4 V vs. SHE. Arrows indicate appendages extending from cells to the electrode surface.

Figure S9. Sodium dodecyl sulfate-polyacrylamide gel electrophoresis (SDS-PAGE) analysis of cell wall fractions isolated from exponential- and stationary-phase CT1112 cells. Gels were stained with Coomassie Brilliant Blue (CBB) to visualize total proteins or with 3,3′-diaminobenzidine (DAB) in the presence of H_2_O_2_ to detect redox-active proteins. No redox-active proteins were detected in the extracted cell wall fractions from either growth phase.

Figure S10. Proposed pathways for the dissipation of excess reducing equivalents in CT1112 cells mediated by extracellular electron transfer (EET). In the first pathway, an electron-bifurcating [FeFe] hydrogenase co-oxidizes NADH and reduced ferredoxin generated by pyruvate:ferredoxin oxidoreductase, coupling their oxidation to proton reduction and H₂ production. In the second pathway, a partial Complex I–like module comprising NuoF, NuoB/C, and membrane-associated NuoK/H/L subunits is depicted. Genes required for ubiquinone or menaquinone biosynthesis are absent from the genome, and electrons downstream of NuoF and NuoB/C are proposed to be directed toward alternative intracellular or extracellular electron carriers. Gene numbers correspond to the original locus tags listed in Table S2.

Table S1. List of proteins predicted to localize to the cell wall by PSORTb.

| **Accession** | **Old locus** | **Definition** |
| --- | --- | --- |
| WP_004623389.1 | *CTER_0134* | metallophosphoesterase |
| WP_004623558.1 | *CTER_0192* | S-layer homology domain-containing protein |
| WP_004623504.1 | *CTER_0219* | S-layer homology domain-containing protein |
| WP_004623596.1 | *CTER_0300* | RHS repeat-associated core domain-containing protein |
| WP_004623839.1 | *CTER_0472* | TlpA family protein disulfide reductase |
| WP_004623838.1 | *CTER_0486* | PA14 domain-containing protein |
| WP_004623934.1 | *CTER_0624* | cadherin-like beta sandwich domain-containing protein |
| WP_004624115.1 | *CTER_0758* | peptide ABC transporter substrate-binding protein |
| WP_004624285.1 | *CTER_0768* | ABC transporter substrate-binding protein |
| WP_004624102.1 | *CTER_0795* | S-layer homology domain-containing protein |
| WP_004624205.1 | *CTER_0847* | nickel ABC transporter substrate-binding protein |
| WP_004624365.1 | *CTER_0945* | Ig-like domain-containing protein |
| WP_004624385.1 | *CTER_0978* | peptide ABC transporter substrate-binding protein |
| WP_004624482.1 | *CTER_0980* | S-layer homology domain-containing protein |
| WP_004624333.1 | *CTER_0988* | S-layer homology domain-containing protein |
| WP_004624627.1 | *CTER_1153* | alpha amylase family protein |
| WP_004624699.1 | *CTER_1212* | polysaccharide lyase family 8 super-sandwich domain-containing protein |
| WP_004625007.1 | *CTER_1462* | non-ribosomal peptide synthetase |
| WP_004624947.1 | *CTER_1539* | family 16 glycoside hydrolase |
| WP_004624793.1 | *CTER_1546* | ABC transporter substrate-binding protein |
| WP_004625360.1 | *CTER_1685* | peptide ABC transporter substrate-binding protein |
| WP_004625253.1 | *CTER_1735* | S-layer homology domain-containing protein |
| WP_004625424.1 | *CTER_1914* | Kelch repeat-containing protein |
| WP_004625560.1 | *CTER_1991* | stage II sporulation protein D |
| WP_004625505.1 | *CTER_2004* | BslA/BslB family hydrophobin |
| WP_004625584.1 | *CTER_2005* | S-layer homology domain-containing protein |
| WP_004625847.1 | *CTER_2258* | SpoIID/LytB domain-containing protein |
| WP_004625833.1 | *CTER_2340* | ABC transporter substrate-binding protein |
| WP_004625969.1 | *CTER_2432* | S8 family serine peptidase |
| WP_004625966.1 | *CTER_2476* | family 10 glycosylhydrolase |
| WP_004625909.1 | *CTER_2496* | carboxypeptidase regulatory-like domain-containing protein |
| WP_004626119.1 | *CTER_2515* | TlpA family protein disulfide reductase |
| WP_004626370.1 | *CTER_2615* | S-layer homology domain-containing protein |
| WP_004626772.1 | *CTER_2895* | ABC transporter substrate-binding protein |
| WP_004627197.1 | *CTER_3247* | glycoside hydrolase family 15 protein |
| WP_004627771.1 | *CTER_3346* | N-acetylmuramoyl-L-alanine amidase |
| WP_004627621.1 | *CTER_3349* | leucine-rich repeat domain-containing protein |
| WP_004627742.1 | *CTER_3425* | Hsp20/alpha crystallin family protein |
| WP_004627583.1 | *CTER_3427* | C40 family peptidase |
| WP_004627720.1 | *CTER_3428* | N-acetylmuramoyl-L-alanine amidase family protein |
| WP_004627811.1 | *CTER_3499* | S-layer homology domain-containing protein |
| WP_004627864.1 | *CTER_3509* | S-layer homology domain-containing protein |
| WP_004627862.1 | *CTER_3516* | S-layer homology domain-containing protein |
| WP_004628048.1 | *CTER_3655* | S-layer homology domain-containing protein |
| WP_004628138.1 | *CTER_3683* | bifunctional metallophosphatase/5'-nucleotidase |
| WP_004628256.1 | *CTER_3815* | RHS repeat-associated core domain-containing protein |
| WP_004628317.1 | *CTER_3822* | S-layer homology domain-containing protein |
| WP_004628323.1 | *CTER_3848* | S-layer homology domain-containing protein |
| WP_004628742.1 | *CTER_4060* | InlB B-repeat-containing protein |
| WP_004628865.1 | *CTER_4067* | S-layer homology domain-containing protein |
| WP_004628806.1 | *CTER_4085* | S-layer homology domain-containing protein |
| WP_004628995.1 | *CTER_4087* | HYR domain-containing protein |
| WP_004629405.1 | *CTER_4403* | Ig-like domain-containing protein |
| WP_004629526.1 | *CTER_4441* | carbohydrate-binding domain-containing protein |
| WP_004629908.1 | *CTER_4535* | S-layer homology domain-containing protein |
| WP_004629729.1 | *CTER_4595* | N-acetylmuramoyl-L-alanine amidase |
| WP_004629625.1 | *CTER_4675* | peptidoglycan-binding protein |
| WP_004629583.1 | *CTER_4707* | family 10 glycosylhydrolase |
| WP_004630225.1 | *CTER_4729* | S-layer homology domain-containing protein |
| WP_004629636.1 | *CTER_4905* | S-layer homology domain-containing protein |
| WP_004630736.1 | *CTER_5200* | RHS repeat-associated core domain-containing protein |
| WP_004631143.1 | *CTER_5361* | carbohydrate-binding protein |
| WP_004623279.1 | *CTER_5516* | N-acetylmuramoyl-L-alanine amidase family protein |
| WP_004623286.1 | *CTER_5566* | COG1361 S-layer family protein |

Table S2. Genes encoding redox-active proteins potentially involved in redox balancing in CT1112. Asterisks (*) denote gene products predicted to localize to the cytoplasmic membrane.

| **Locus tag** | **Gene** | **Annotation** | **Proposed function** |
| --- | --- | --- | --- |
| *CTER_0018* | *nuoB* | NADH-quinone oxidoreductase subunit NuoB |  |
| *CTER_0657* | *nuoC* | NADH-quinone oxidoreductase subunit C | - Nuo-like subunits potentially involved in NADH oxidation. - The genome lacks genes for (mena)quinone biosynthesis, suggesting the absence of a quinone pool. - Downstream electrons may be routed toward EET or diverted to an unknown electron carrier. |
| *CTER_0658* | *nuoF* | hydrogenase 4 subunit F |  |
| *CTER_0659* |  | hypothetical protein |  |
| *CTER_0660* | *nuoH* | respiratory chain complex I subunit 1 family protein |  |
| *CTER_0661* | *nuoB* | hydrogenase 4 subunit B |  |
| *CTER_3889* | *hypB* | hydrogenase nickel incorporation protein HypB | - Membrane-bound H_2_-oxidizing [NiFe] hydrogenase with H⁺-translocating subunits |
| **CTER_3890* |  | trimeric intracellular cation channel family protein |  |
| *CTER_3891* | *hypA* | hydrogenase maturation nickel metallochaperone HypA |  |
| *CTER_3892* |  | 4Fe-4S binding protein |  |
| *CTER_3893* | *hycE2* | nickel-dependent hydrogenase large subunit |  |
| *CTER_3894* | *nuoC* | NADH-quinone oxidoreductase subunit C |  |
| **CTER_3895* | *nuoB* | NADH-quinone oxidoreductase subunit B family protein |  |
| **CTER_3896* | *nuoH* | respiratory chain complex I subunit 1 family protein |  |
| **CTER_3897* | *nuoL* | NADH-quinone oxidoreductase subunit L |  |
| *CTER_4761* |  | NADH-dependent [FeFe] hydrogenase, group A6 | - Multilocus electron-bifurcating, H_2_-evolving [FeFe] hydrogenase for H_2_ production. - This cluster may enable the cell to dissipate excess reducing equivalents as H_2_. |
| *CTER_4762* | *nuoF* | NADH-quinone oxidoreductase subunit NuoF |  |
| *CTER_4763* |  | (2Fe-2S) ferredoxin domain-containing protein |  |
| *CTER_4764* |  | ATP-binding protein |  |
| *CTER_4765* | *nuoE* | [FeFe] hydrogenase |  |
| *CTER_4846* |  | [Fe-Fe] hydrogenase large subunit C-terminal domain-containing protein |  |
| *CTER_4847* |  | (2Fe-2S) ferredoxin domain-containing protein |  |
| *CTER_4848* |  | [FeFe] hydrogenase, group A |  |
| *CTER_4849* | *nuoF* |  |  |
| *CTER_4850* |  | complex I 24 kDa subunit family protein |  |
